# Potential anti-aging agents suppress the level of constitutive mTOR- and DNA damage- signaling

**DOI:** 10.18632/aging.100521

**Published:** 2012-12-30

**Authors:** H. Dorota Halicka, Hong Zhao, Jiangwei Li, Yong-Syu Lee, Tze-Chen Hsieh, Joseph M. Wu, Zbigniew Darzynkiewicz

**Affiliations:** ^1^ Brander Cancer Research Institute, Department of Pathology, New York Medical College, Valhalla, NY 10595, USA; ^2^ Department of Biochemistry and Molecular Biology, New York Medical College, Valhalla, NY 10595, USA

**Keywords:** H2AX phosphorylation, ROS, ribosomal protein S6, calorie restriction, metformin, rapamycin, 2-deoxyglucose, rapamycin, berberine, vitamin D3, resveratrol, aspirin, replication stress, senescence, cell cycle, 4EBP1

## Abstract

Two different mechanisms are considered to be the primary cause of aging. Cumulative DNA damage caused by reactive oxygen species (ROS), the by-products of oxidative phosphorylation, is one of these mechanisms (ROS concept). Constitutive stimulation of mitogen- and nutrient-sensing mTOR/S6 signaling is the second mechanism (TOR concept). The flow- and laser scanning- cytometric methods were developed to measure the level of the constitutive DNA damage/ROS- as well as of mTOR/S6- signaling in individual cells. Specifically, persistent activation of ATM and expression of γH2AX in untreated cells appears to report constitutive DNA damage induced by endogenous ROS. The level of phosphorylation of Ser235/236-ribosomal protein (RP), of Ser2448-mTOR and of Ser65-4EBP1, informs on constitutive signaling along the mTOR/S6 pathway. Potential gero-suppressive agents rapamycin, metformin, 2-deoxyglucose, berberine, resveratrol, vitamin D3 and aspirin, all decreased the level of constitutive DNA damage signaling as seen by the reduced expression of γH2AX in proliferating A549, TK6, WI-38 cells and in mitogenically stimulated human lymphocytes. They all also decreased the level of intracellular ROS and mitochondrial trans-membrane potential ΔΨm, the marker of mitochondrial energizing as well as reduced phosphorylation of mTOR, RP-S6 and 4EBP1. The most effective was rapamycin. Although the primary target of each on these agents may be different the data are consistent with the downstream mechanism in which the decline in mTOR/S6K signaling and translation rate is coupled with a decrease in oxidative phosphorylation, (revealed by ΔΨm) that leads to reduction of ROS and oxidative DNA damage. The decreased rate of translation induced by these agents may slow down cells hypertrophy and alleviate other features of cell aging/senescence. Reduction of oxidative DNA damage may lower predisposition to neoplastic transformation which otherwise may result from errors in repair of DNA sites coding for oncogenes or tumor suppressor genes. The data suggest that combined assessment of constitutive γH2AX expression, mitochondrial activity (ROS, ΔΨm) and mTOR signaling provides an adequate gamut of cell responses to evaluate effectiveness of gero-suppressive agents.

## INTRODUCTION

The cumulative DNA damage caused by reactive oxygen species (ROS), by-products of oxidative phosphorylation, for long time has been considered to be a key factor contributing both to cell aging as well as predisposing to neoplastic transformation [1-12]. Oxidative DNA damage generates significant number of DNA double-strand breaks (DSBs), the potentially deleterious lesions. DSBs can be repaired either by the homologous recombination or nonhomologous DNA-end joining (NHEJ) mechanism. Recombinatorial repair which uses newly replicated DNA as a template restores DNA rather faithfully. It can take place however when cells have already the template, namely during late-S and G_2_ phase. In the cells that lack a template (G_1_, early-S) DNA repair relies on the NHEJ which is error-prone due to a possibility of a deletion or rearrangement of some base pairs [13-17]. If the erroneously repaired DSBs are at sites of oncogenes or tumor suppressor genes this may result in somatic mutations that predispose cell to oncogenic transformation. Oxidative damage of telomeric DNA may lead to dysfunction of telomeres thereby driving cells to undergo replicative senescence [18-30].

Whereas DNA damage induced by endogenous (and exogenous) oxidants may indeed significantly contribute to cancer development its role as being the key factor accountable either for cellular or organismal aging is debatable [31-40]. There is growing body of evidence in support of the notion that the primary culprit of aging is the constitutive stimulation of the mitogen- and nutrient-sensing signaling pathways. Activation of these pathways enhances translation, leads to cell growth in size/mass and ultimately results in cell hypertrophy and senescence. Among these culprit pathways the mammalian target of rapamycin (mTOR) and its downstream target S6 protein kinase (S6K) play the key role [41-49]. Constitutive replication stress likely resulting from the ongoing oxidative DNA damage when combined with activation of mTOR/S6K appears to be the driving force leading to aging and senescence both at the cellular as well as organismal level [43-51].

We have recently reported that constitutive DNA damage signaling (CDDS) observed in the untreated normal or tumor cells, assessed as the level of expression of histone H2AX phosphorylated on Ser139 (γH2AX) and of activated (Ser1981 phosphorylated) *Ataxia Telangiectasia mutated* protein kinase (ATM), is an indication of the ongoing DNA damage induced by endogenous ROS [52-55]. These phosphorylation events were detected with phospho-specific antibodies (Ab) and measured in individual cells by flow- or laser scanning- cytometry. Using this approach we have assessed several agents reported to have anti-oxidant and DNA-protective properties with respect to their ability to attenuate the level of CDDS [52-58]. In the present study we test effectiveness of several reported anti-aging modalities to attenuate the level of CDDS in individual TK6 and A549 tumor cell lines as well as in WI-38 and mitogenically stimulated normal lymphocytes.

In parallel, we also assess their effect on the level of constitutive state of activation of the critical mTOR downstream targets. Specifically, using phospho-specific Abs detecting activated status of ribosomal protein S6 (RP-S6) phosphorylated on Ser235/236 we measure effectiveness of these gero-suppressive agents along the mTOR/S6K signaling. We have also tested effects of these agents on the level of endogenous reactive oxidants as well as mitochondrial electrochemical potential ΔΨm. The following agents, reported as having anti-aging and/or chemopreventive properties, were chosen in the present study: 2-deoxy-D-glucose (2dG) [59-62], metformin (MF) [63-71], rapamycin (RAP) [72-80], berberine (BRB) [81-85], vitamin D3 (Vit. D3) [86- 91], resveratrol (RSV) [92-97] and acetylsalicylic acid (aspirin) (ASA) [98-103].

## RESULTS

**Figure 1:**
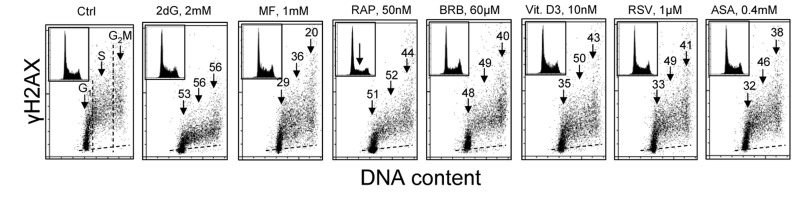
Effect of exposure of TK6 cells to different presumed anti-aging drugs on the level of constitutive expression of γH2AX Exponentially growing TK6 cells were untreated (Ctrl) or treated with the respective agents for 24 h at concentrations as shown. Expression of γH2AX in individual cells was detected immunocytochemically with the phospho-specific Ab (AlexaFluor647), DNA was stained with DAPI; cellular fluorescence was measured by flow cytometry. Based on differences in DNA content cells were gated in the respective phases of the cell cycle, as marked by the dashed vertical lines. The percent decrease in mean fluorescence intensity of the treated cells in particular phases of the cell cycle, with respect to the respective untreated controls, is shown above the arrows. Inserts present DNA content frequency histograms from the individual cultures. The dashed skewed lines show the background level, the mean fluorescence intensity of the cells stained with secondary Ab only.

Fig. 1 illustrates the effect of exposure of human lymphoblastoid TK6 cells for 24 h to the investigated presumed anti-aging agents on the level of constitutive expression of γH2AX. Consistent with our prior findings [52-54] the expression γH2AX in S and G_2_M cells is distinctly higher than in the cells of G_1_ phase. This is the case for both, the untreated (Ctrl) cells as well as the cells treated with these agents. It is also apparent that exposure of cells to each of the studied drugs led to the decrease in expression of γH2AX in all phases of the cell cycle. In most treated cells, however, the decline in the mean expression γH2AX was somewhat more pronounced in the S- compared to G_1_ - or G_2_M- phase cells. Analysis of DNA content frequency histograms reveals that the 24 h treatment with most of the drugs had no effect on the cell cycle distribution. The exception are the cells treated with 50 nM RP which show about 50% reduction in frequency of cells in S and G_2_M which would indicate partial cells arrest in G_1_ phase of the cell cycle. It should be noted that exposure of cells to these agents for 4 h led to rather minor (<15%) decrease in expression of γH2AX whereas the treatment for 48 h had similar effect as for 24 h (data not shown).

**Figure 2:**
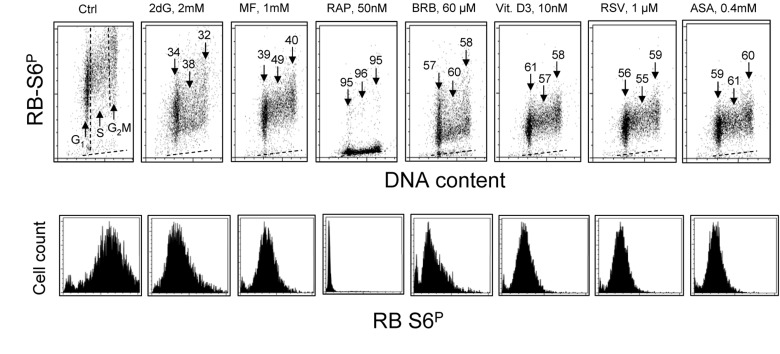
Effect of treatment of TK6 cells with different presumed anti-aging drugs for 4 h on the level of constitutive phosphorylation of ribosomal protein S6 (RP-S6) Exponentially growing TK6 cells were untreated (Ctrl) or treated with the respective agents at concentrations as shown. Phosphorylation status of ribosomal S6 protein was detected immunocytochemically with the phospho-specific Ab (AlexaFluor647), DNA was stained with DAPI; cellular fluorescence was measured by flow cytometry. **Top panels:** Based on differences in DNA content cells were gated in the respective phases of the cell cycle, as marked by the dashed vertical lines (Ctrl). The percent decrease in mean fluorescence intensity of the treated cells in particular phases of the cell cycle, with respect to the to the same phases of the untreated cells, is shown above the arrows. The dashed skewed lines show the background level, the mean fluorescence intensity of the cells stained with secondary Ab only. **Bottom panels:** Single parameter frequency histograms showing expression of phosphorylated ribosomal S6 protein (RB-S6^P^) in all (G_1_+S+G_2_M) cells of the respective cultures.

The effect of exposure of TK6 cells to the investigated gero-suppressive agents on state of phosphorylation of ribosomal S6 protein is shown in Fig. 2. Unlike expression of γH2AX the level of phosphorylation of RP-S6 shows no significant cell cycle phase-related differences, neither in control nor in the treated cultures. Somewhat higher expression of RP-S6^P^ in S- and G_2_M- compared to G_1_- cells is proportional to an overall increase in cell size during cell cycle progression. It is quite evident however that the treatment with each of these anti-aging agents led to a decrease in the level of phosphorylation of S6 protein. The most dramatic decrease (>95%) was seen in the cells treated with RAP. The cells treated with 2dG showed the smallest (32-38%) decrease. There was no evidence that treatment of TK6 cells with all these drugs for 4 h had any distinct effect on the cell cycle progression as detected by analysis of DNA content frequency histograms (not shown).

**Figure 3:**
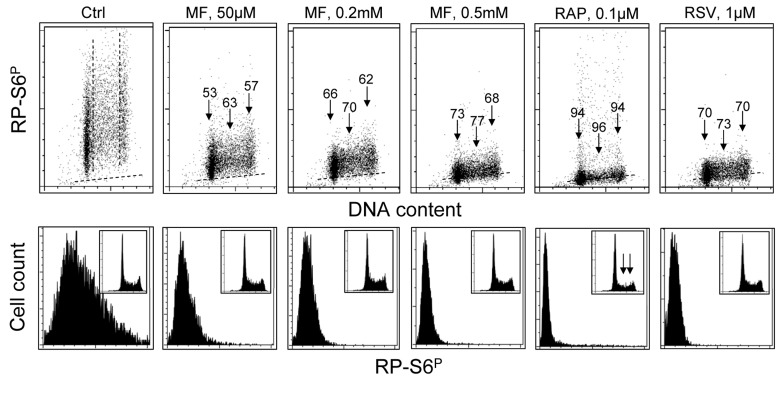
Effect of treatment of TK6 cells with MF, RAP or RSV for 24 h on the level of constitutive phosphorylation of S6 protein TK6 cells were untreated (Ctrl) or treated with different concentrations of MF as well as with RAP or RSV for 24 h. Phosphorylation status of S6 was assessed as described in legend to Fig. 2. **Top panels:** The percent decrease in mean fluorescence intensity of the drug-treated cells in particular phases of the cell cycle is shown above the arrows. **Bottom panels:** Frequency histograms showing expression of RP-S6P in all cells of the respective cultures. Insets show cellular DNA content histograms of cells in these cultures.

Fig. 3 presents the effect of exposure of TK6 cells to MF, RAP or RSV at somewhat lower concentration for 24 h on the level of expression of RP-S6^P^. Compared with cells exposed for 4 h (Fig. 1) the effect of MF, even at the lower concentration (50 μM), was more pronounced after 24 h. Also, after that time of exposure, more pronounced was the effect of RAP and RSV.

Analysis of the DNA content frequency histograms indicates that neither 50 – 500 μM MF nor RSV had an effect on the cell cycle progression. However, exposure to 0.1 μM RAP (similar to 50 nM, see Fig. 1) resulted in about 50% decrease in frequency of S and G_2_M cells (insets, marked by arrows).

**Figure 4:**
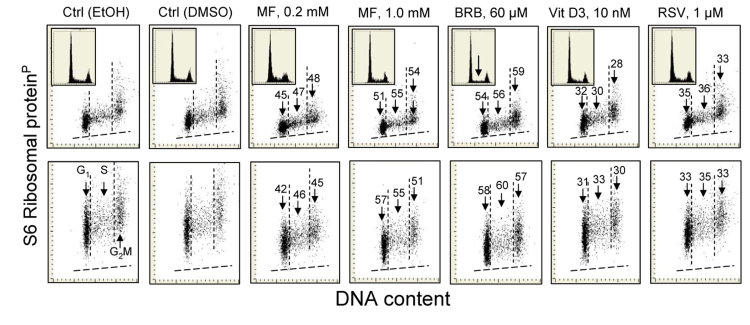
Reduction of the level of constitutive expression of RP-S6P in A549 cells exposed to MF, BRB, Vit. D3 or RSV for 24 h Exponentially growing in chamber slides A549 cells, were treated with the respective agents and their fluorescence was measured the laser scanning cytometry (LSC)^75^. Top panels show RP-S6^P^ immunofluorescence integrated over the nuclei (reporting expression of RP-S6^P^ in the cytoplasm located over and below the nucleus); bottom panels present RP-S6^P^ immunofluorescence integrated over the cytoplasm aside of the nucleus. The percent decrease in expression of RP-S6^P^ in cells in particular phases of the cell cycle (mean values) is shown above the arrows. Because stock solutions of some of these agents were made in DMSO, other in MeOH or EtOH, the equivalent quantities of these solvents were included in the respective control culture and the percent decrease shown in the panels refers to the decrease compared to these controls shown are the cells from EtOH and DMSO containing controls. The insets present DNA content frequency histograms from the respective cultures.

The effect of some of these gero-suppressive drugs was also studied on human pulmonary adenocarcinoma A549 cells (Fig. 4). These cells grow attached and their fluorescence intensity was measured by imaging cytometry (laser scanning cytometer; LSC) [104]. The decrease in expression of RP-S6^P^ was seen in the cells treated with each of the drugs. The effect was essentially of similar degree whether measured in cytoplasm over- and underlying the nucleus (Fig. 4 top panels) or in the cytoplasm at the nuclear periphery (bottom panels). The most pronounced decrease was induced by BRB. Also affected was the cell cycle progression, as evidenced by the decline in frequency of S-phase cells on the DNA histogram in BRB treated cells (inset, marked by arrow).

**Figure 5:**
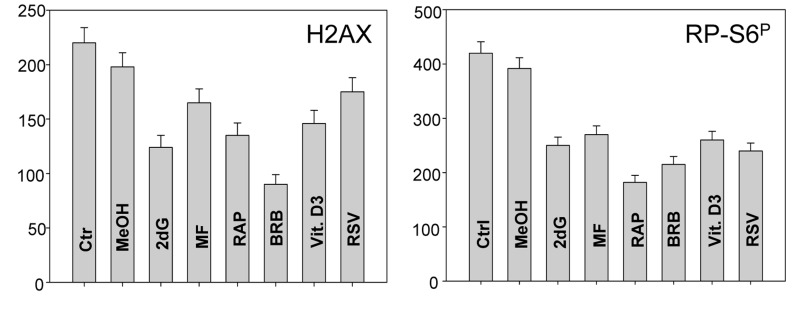
Effect of treatment of WI-38 cells with 2dG, MF, RAP, BRB, Vit. D3 or RSV for 24 h on the level of constitutive expression of γH2AX (left panel) and RP-S6^P^ (right panel) Exponentially growing cells, were treated with the respective agents at concentrations as shown in Figs. 1 and 2, RP-S6^P^ was detected immunocytochemically and cell fluorescence was measured with the laser scanning cytometry (LSC). The bar graphs present the mean fluorescence intensity measured as an integral over the nucleus (γH2AX) or over cytoplasm (PR-S6^P^).

**Figure 6:**
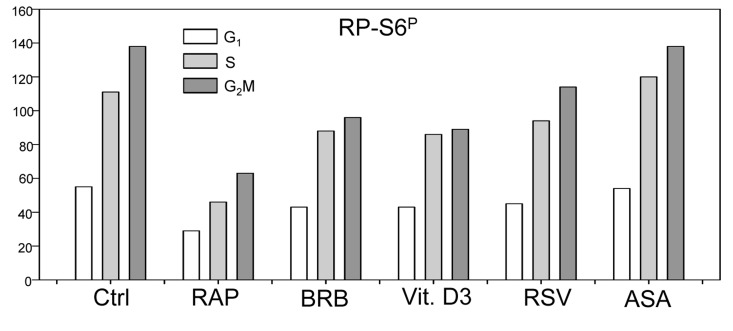
Effect of treatment of mitogenically stimulated human lymphocytes with RAP, BRB, Vit. D3, RSV or ASA for 4 h on the level of constitutive expression of RP-S6^P^ Peripheral blood lymphocytes were mitogenically stimulated with phytohemagglutinin (PHA) for 72 h, the cells were then treated with the respective drugs at concentrations as shown in Figs. 1 and 2 for 4 h, RP-S^6^ wasdetected immunocytochemically and cellular fluorescence measured by flow cytometry. The bar graphs present the mean values (+SD) of RP-S6^P^ immunofluorescence for G_1_, S and G_2_M cell subpopulations identified by differences in DNA content (intensity of DAPI fluorescence).

In addition to tumor cell lines we have also tested effects of the presumed gero-suppressive agents on non-tumor cells. Fig. 5 illustrates their effect on the WI-38 cells and Fig. 6 on mitogenically-stimulated human lymphocytes. A decrease in expression of γH2AX was observed in WI-38 cells treated with each of the tested agents, the most pronounced reduction (>50%) showed cells treated with BRB while the least affected (<10%) were cells growing in the presence of RSV. A reduction in the level of phosphorylated RB-S6 was also evident in WI-38 cells exposed to each of these agents, the most pronounced (>50%) after treatment with RAP. Because stock solutions of some of these agents were made in DMSO or MeOH equivalent quantities of these solvents were included in the respective control cultures. A minor suppressive effect of MeOH on expression of γH2AX and RB-S6^P^ was observed (Fig. 5). Likewise, DMSO exerted also minor (~5%) but repeatable suppressive effect (not shown). As is evident RAP, BRB, Vit. D3 and RSV reduced the level of RP-S6^P^ in mitogenically stimulated human lymphocytes, in all phases of the cell cycle, while the effect of ASA on these cells was minimal (Fig. 6).

**Figure 7:**
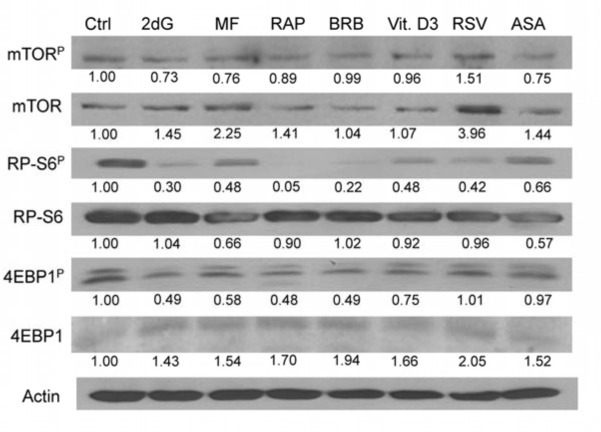
Effect of the studied gero-preventive agents on constitutive level of expression of mTOR-Ser2448^P^, RP-S6-Ser235/236^P^ and 4EBP1-Ser65^P^ and their corresponding unphosphorylated forms in TK6 cells, detected by western blotting TK6 cells were exposed to the studied agents at concentrations as shown in Figs 1 and 2 for 4 h. The protein expression level were determined by western blot analysis and the intensity of the specific immunoreactive bands were quantified by densitometry and normalized to actin (loading control). The numbers indicate the n-fold change in expression of the respective phospho-proteins in the drug-treated cultures with respect to the untreated cells (Ctrl).

To confirm the findings obtained by the flow- and laser scanning- cytometry based on measurement of individual cells we assessed effects of the gero-suppressive agents by measurement mTOR signaling in bulk, by western blotting. In this experiment, having available phospho-specific Abs that detect phosphorylation of mTOR, RP-S6 and the eukaryotic translation initiation factor 4E-binding protein (4EBP1) applicable to western blotting (not yet available for cytometry) we have been able to test effects of the studied gero-preventive agents on the level of constitutive phosphorylation of these proteins as well. As is evident in Fig. 7 and Table 1 exposure of TK6 cells to the gero-preventive agents lowered the level of phosphorylation status of mTOR, as well as its downstream targets RP-S6 and 4EBP1. The most pronounced effect was seen in the case of RAP, BRB and 2dG which lowered expression of RP-S6^P^ by 95%,78 and 70%, respectively. RAP, BRB and 2dG were also quite effective in lowering the level of 4EBP1^P^, by 52%, 51% and 51%.

**Table 1:** Effect of the studied gero-preventive agents on constitutive level of expression of mTOR-Ser2448^P^, RP-S6-Ser235/236^P^ and 4EBP1-Ser65^P^ and their corresponding unphosphorylated forms, detected by western blotting (Fig. 7)

Agent	Ctrl	2dG	MF	RAP	BRB	Vit. D3	RSV	ASA
mTOR^P^	1.00	0.73	0.76	0.89	0.99	0.96	1.51	0.75
m-TOR	1.00	1.45	2.25	1.41	1.04	1.07	3.96	1.44
**RATIO**	**1.00**	**0.50**	**0.34**	**0.63**	**0.95**	**0.90**	**0.38**	**0.52**
RP-S6^P^	1.00	0.30	0.48	0.05	0.22	0.48	0.42	0.66
S6	1.00	1.04	0.66	0.9	1.02	0.92	0.96	0.57
**RATIO**	**1.00**	**0.29**	**0.73**	**0.06**	**0.22**	**0.52**	**0.44**	**1.16**
4EBP1^P^	1.00	0.49	0.58	0.48	0.49	0.75	1.01	0.97
4EBP1	1.00	1.43	1.54	1.7	1.94	1.66	2.05	1.52
**RATIO**	**1.00**	**0.38**	**0.38**	**0.28**	**0.25**	**0.45**	**0.45**	**0.64**

The numbers indicate the change in expression of the respective proteins in the dug-treated cultures with respect to the untreated ones. Densitometric quantification of phosphorylated and total proteins for mTOR, RP-S6 and 4EBP1 are presented as the ***ratio*** of actin-normalized phosphorylated to total protein level of expression (**Bold font**).

Most interesting, however, were the results reporting effects of the studied drugs on the total mTOR, RP-S6 and 4EBP1 protein content and on the ratios of the phosphorylated protein fractions to the total content of the respective proteins (Table 1). These data show that exposure of cells to each drug led to a distinct up-regulation of mTOR and 4EBP1 expression. This was not the case of RP-S6, which, with an exception of 2dG and BRB, showed a minor decline. However, compared with the apparent increase of total proteins content, the level of the phosphorylated fractions of the respective proteins was more severely reduced. This over-compensated the upregulation and is expressed as the reduction of the ratio of phosphorylated to total content of the respective proteins. In the case of RP-S6 and 4EBP1, the downstream effectors of mTOR and the agents directly affecting the translation rate, the most effective was BRB and RAP, reducing proportion of the phosphorylated to total protein content by 75% and 72% respectively (Table 1).

**Figure 8:**
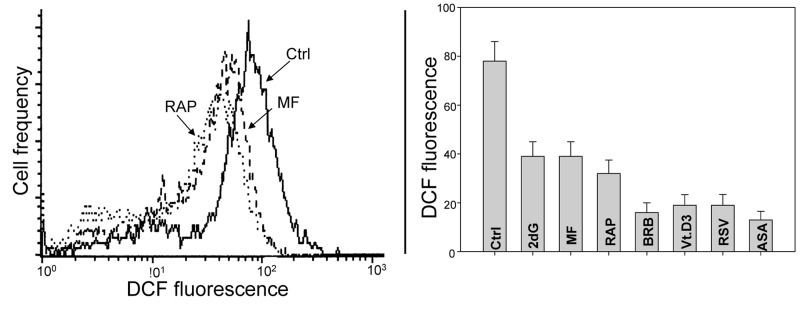
Effects of the studied gero-preventive agents on the intercellular level of ROS TK6 cells, untreated (Ctrl) or treated for 24 h with the investigated agents, were exposed for 30 min to H_2_DCF-DA and their fluorescence intensity was measured by flow cytometry. The cell-permeant non-fluorescent H_2_DCF-DA upon cleavage of the acetate moiety by intercellular esterases and oxidation by ROS is converted to strongly fluorescent DCF and thus reports the ROS abundance. Left panel shows the frequency histograms of the untreated (Ctrl) as well MF and RAP-treated cells (note exponential scale of the DCF fluorescence). Right panel presents the mean values (+SD) of DCF fluorescence of the untreated (Ctrl) and treated cells.

In other set of experiments we assessed the effect of the studied gero-preventive agents on the level of endogenous ROS. As is evident in Fig. 8 exposure of TK6 cells to each of these agents led to a marked reduction of cells ability to oxidize H_2_DCF-DA; its oxidation by ROS results in formation of the strongly fluorescent DCF which is considered to be a marker of ROS abundance. In this respect more effective appeared to be BRB, Vit. D3, RSV and ASA compared to RAP, MF or 2dG.

**Figure 9:**
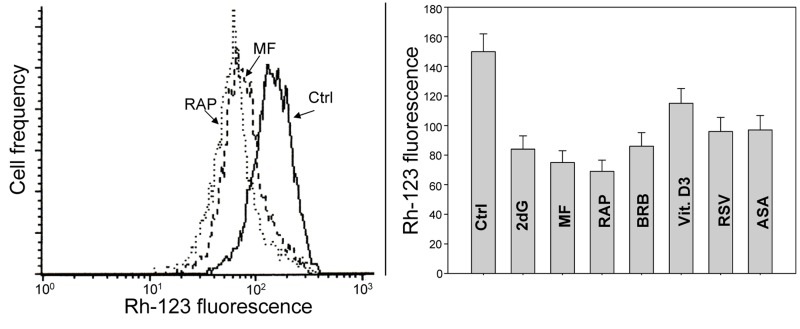
Effect of the studied gero-preventive agents on the mitochondrial transmembrane potential (ΔΨm) TK6 cells, untreated (Ctrl) or treated for 24 h with the investigated agents were exposed for 30 min to the mitochondrial probe rhodamine 123 (Rh-123) and their fluorescence intensity was measured by flow cytometry. Left panel shows the frequency histograms of the untreated (Ctrl) as well MF and RAP-treated cells (note exponential scale of the DCF fluorescence). Right panel presents the mean values (+SD) of Rh-123 fluorescence of the investigated cells.

Fig. 9 illustrates changes in electrochemical transmembrane potential of mitochondria detected by cells capability to accumulate the mitochondrial probe rhodamine 123 (Rh-123) in TK6 cells treated with the investigated gero-preventive agents. The data show a reduction in the ability to accumulate Rh-123 in cells treated with each of these agents, the most pronounced in the case of treatment with RAP.

## DISCUSSION

In the prior studies we have already observed that MF at concentrations 0.1 mM – 20 mM [55] and Vit. D (2 nM - 10 nM) [56]effectively reduced constitutive level of H2AX-Ser139 and ATM-Ser1981 phosphorylation. In the present study all seven agents, all reportedly having anti-aging and/or chemopreventive properties, including MF and Vit D3[59-103], have been tested with respect of their ability to affect both the level of constitutive DNA damage signaling as monitored γH2AX expression as well as constitutive level of phosphorylation of ribosomal S6 protein (RP-S6^P^). The data show that each of the drugs reduced both, the level of phosphorylation of both H2AX on Ser139 and RP-S6 on Ser235/236. RP-S6, a component of the 40S ribosomal subunit and the most downstream effector of mTOR signaling, is directly involved in regulation of translation [46] and considered to be a determinant of cell size [105,106]. As is evident from the western blotting data (Fig. 7) with an exception of RSV all the studied drugs reduced also the level of phosphorylation of mTOR, RP-S6 and 4EBP1. The latter protein is also considered to be a critical regulator of translation and cell size determinant [105-108].

Analysis of the mTOR vs. mTOR^P^, RP-S6 vs. RP-S6^P^ and 4EBP1 vs. 4EBP1^P^ revealed up-regulation of mTOR and 4EBP1 in cells treated with each of the studied drugs (Table 1). The increase of total content of these proteins was overcompensated by the reduction in the extent of their phosphorylation, which led to decrease in the ratios of mTOR^P^/mTOR, RP-S6^P^/RP-S6 and 4EB1^P^/4EB1. The upregulation of these proteins was unexpected but it suggests that the reduction of their phosphorylation status by the studied drugs may trigger compensatory synthesis (or reduced turnover rate) that leads to increase in their content. The distinctly reduced ratios of mTOR^P^/mTOR, RP-S6^P^/RP-S6 and 4EB1^P^/4EB1, however, may provide a novel biomarker useful to assess the potential mTOR-inhibitory activities that relate to reduction of translation rate, cell size and thus may be of value in assessing anti-aging properties of the studied agents.

The reduction of RP-S6 phosphorylation by each of the gero-suppressive drugs was presently observed in all types of the cells, including tumor TK6 (Fig. 1-3,7) and A549 (Fig. 4) cell lines as well as in normal WI-38 (Fig. 5) and mitogenically stimulated human lymphocytes (Fig. 6). The results obtained by flow and laser scanning cytometry were confirmed by measurement in bulk, by western blotting. The western blotting approach allowed us also to measure phosphorylation level of mTOR and 4EBP1 to which the commercially available phospho-specific Abs are not fully applicable for flow or laser scanning cytometry. The cytometric approach has an advantage that it provides information regarding the cell cycle phase specificity of expression of γH2AX or RP-S6. Furthermore, the cytometric approach has no potential risk of an artifact that the level of phosphorylation of the studied proteins may be altered as a result of disruption of cell integrity in preparation for blotting, which may provide contact of these proteins with active phosphatases and kinases. We observed, for example that when inhibitors of phosphatases were not rigorously used during cell preparation for western blotting the results (not shown) were entirely different than in Fig. 7.

According to the mTOR concept of the mechanism of aging the observed reduction of the level of phosphorylation of mTOR, 4EBP1 and RP-S6 by the studied agents would be consistent with their reported anti-aging properties. The gero-preventive mechanism of these agents thus would be similar to that of the calorie restriction which was definitely proven to extent life span of a variety of organisms [94-97,109].

Parallel to the reduction of constitutive mTOR/S6 signaling each of the investigated gero-suppressive agents also reduced CDDS, as seen by the decline in γH2AX expression (Fig. 1). This corresponding response to these agents, concurrently by both the DNA damage- and mTOR- signaling pathways, suggests on mechanistic association between these two pathways that may converge on the aging-related processes. One of the mechanisms linking these pathways is straightforward as it may involve a decrease of intensity of oxidative phosphorylation in mitochondria. Namely, because the declined translation rate requires less energy the intensity of oxidative phosphorylation that generates ROS is reduced which results in attenuation of CDDS. Consistent with this mechanism is our prior observation that exposure of lymphocytes to Vit. D3 led to a three-fold decline in abundance of ROS [55]. Likewise, treatment of TK6 cells with MF resulted in a significant decrease in the level of ROS [56]. In the present study we confirmed these earlier findings as we observed that all studied drugs markedly lowered abundance of ROS in TK6 cells (Fig. 8). Accordingly, mitogenic stimulation of lymphocytes known to dramatically enhance transcription and translation rates [110,111] also was seen to boost production of ROS and augment CDDS [112]. There are numerous linkages connecting DNA damage response with mTOR/RP-S6 pathways, primarily involving p53 signaling [113-118]. Of interest, and confirming the involvement of mitochondrial pathways in response to the studied gero-preventive agents, is also the observation that exposure of cells to each of them resulted in a decreased mitochondrial transmembrane potential (ΔΨm). The latter was detected by reduced cells capability to bind rhodamine 123 (Fig. 9), the probe known to be the marker of energized mitochondria [119-121].

Unlike constitutive phosphorylation of RP-S6 which was unrelated to the cell cycle phase, H2AX phosphorylation was cell cycle phase specific, distinctly higher in S- and G_2_M- than in G_1_- cells. This suggests that DNA replication stress may be a contributing factor to the observed CDDS. DNA lesions resulting from oxidative DNA damage caused by endogenous ROS could be responsible for the replication stress. As mentioned in the Introduction constitutive replication stress when concurrent with mTOR/S6K signaling is considered to be the predominant factor leading to aging and senescence. Thus, the present data that show that the investigated gero-preventive drugs suppress both, the mTOR/RP-S6 signaling and CDDS, would be consistent with the mechanism that involves attenuation of DNA replication stress.

Whereas mTOR/S6 signaling is the primary cause of aging and induction of premature cell senescence the DNA damage by reactive oxidants, since it induces DSBs which cannot always be faithfully repaired, predisposes to neoplastic transformation [1-7]. It is expected therefore that the anti-aging agents that reduce CDDS would have cancer preventive properties as well. Indeed such chemo-preventive properties have been described for each of the presently investigated drugs [122-128]. The present data indicate that the combined analysis of: (i) CDDS measured by γH2AX expression, (ii) mitochondria activity (ROS, ΔΨm) and (iii) mTOR signaling (mTOR, S6K,4EBP1 phosphorylation) in individual cells [129] may provide an adequate gamut of cell responses to evaluate potential gero- or chemo- preventive properties of suspected agents.

## MATERIALS AND METHODS

### Cells, Cell Treatment

Human lung carcinoma A549 cells, diploid lung WI-38 fibroblasts and lympho-blastoid TK6 cells were obtained from American Type Culture Collection (ATCC CCL-185, Manassas, VA). Human peripheral blood lymphocytes were obtained by venipuncture from healthy volunteers and isolated by density gradient centrifugation. A549 cells were cultured in Ham's F12K, TK6, WI-38 and lymphocytes were cultured in RPMI 1640 with 2 mM L-glutamine, 1.5 g/L sodium bicarbonate and 10% fetal bovine serum (GIBCO/Invitrogen, Carlsbad, CA). Adherent A549 and WI-38 cells were grown in dual-chambered slides (Nunc Lab-Tek II), seeded with 10^5^ cells/ml suspended in 2 ml medium per chamber. TK6 cells and lymphocytes were grown in suspension; lymphocyte cultures were treated with the polyvalent mitogen phytohemaglutinin (Sigma /Aldrich; St Louis, MO) as described [36]. MF (1,1-dimethylbiguanide) was obtained from Calbiochem, La Jolla, CA, 2dG, RAP, BRB, RSV and ASA from Sigma-Aldrich. The active form of vitamin D3 (1,25-dihydroxyvitamin D3) was kindly provided by Dr Milan Uskokovic [56]. Stock solutions of some of these agents were prepared either in DMSO, MeOH or EtOH as indicated by the vendor. The cells, during exponential phase of growth were treated with these agents, at concentrations and for duration as indicated in the figures or figure legends. Respective control cultures were treated with the equivalent volumes of solvents used for stock solutions. After exposure to the gero-preventive agents the cells were rinsed with phosphate buffered salt solution (PBS) and fixed in 1% methanol-free formaldehyde (Polysciences, Warrington, PA) for 15 min on ice. The cells were then transferred to 70% ethanol and stored at -20 ^o^C for up to 3 days until staining.

### Immunocytochemical Detection of γH2AX and RP-S6^P^

After fixation the cells were washed twice in PBS and with 0.1% Triton X-100 (Sigma-Aldrich) in PBS for 15 min and with a 1% (w/v) solution of bovine serum albumin (BSA; Sigma-Aldrich in PBS for 30 min to suppress nonspecific antibody (Ab) binding. The cells were then incubated in 1% BSA containing a 1:300 dilution of phospho-specific (Ser139) γH2AX mAb (Biolegend, San Diego, CA) and/or with a 1: 200 dilution of phosphospecific (Ser235/236) RP-S6 Ab (Epitomics, Burlingame, CA) at 4^o^C overnight. The secondary Ab was tagged with AlexaFluor 488 or 647 fluorochrome (Invitrogen/Molecular Probes, used at 1:100 dilution in 1% BSA). The incubation was at room temperature for 45 min. Cellular DNA was counterstained with 2.8 μg/ml 4,6-diamidino-2-phenylindole (DAPI; Sigma-Aldrich) at room temperature for 15 minutes. Each experiment was performed with an IgG control in which cells were labeled only with the secondary AlexaFluor 488 Ab, without primary Ab incubation to estimate the extent of nonspecific adherence of the secondary Ab to the cells. The fixation, rinsing and labeling of A549 and WI-38cells was carried out on slides, and lymphocytes and TK6 cells in suspension. Other details have been described previously [53-56].

### Detection of ROS and Mitochondrial Transmembrane Potential ΔΨm

Untreated cells as well as the gero-protective agents drugs-treated TK6 cells were incubated 60 min with 10 μM 2',7'-dihydrodichlorofluorescein- diacetate (H_2_DCF-DA) (Invitrogen/Molecular Probes) at 37°C. Cellular green fluorescence was then measured by flow cytometry. Following oxidation by ROS and peroxides within cells the non-fluorescent substrate H_2_DCF-DA is converted to the strongly fluorescent derivative DCF [97]. Mitochondrial potential ΔΨm was assessed by exposure of cells in tissue culture to 1 μM rhodamine 123 (Rh-123; Invitrogen/Molecular Probes) for 30 min prior to measurement of their fluorescence.

### Analysis of Cellular Fluorescence

*A549 and WI-38 cells:* Cellular immunofluorescence representing the binding of the respective phospho-specific Abs as well as the blue emission of DAPI stained DNA was measured by Laser Scanning Cytometry (LSC) [131] (iCys; CompuCyte, Westwood, MA) utilizing standard filter settings; fluorescence was excited with 488-nm argon, helium neon (633 nm) and violet (405 nm) lasers. Intensities of maximal pixel and integrated fluorescence were measured and recorded for each cell. At least 3,000 cells were measured per sample. Gating analysis was carried out as described in Figure legends. *TK6cells and lymphocytes:* Intensity of cellular fluorescence was measured using a MoFlo XDP (Beckman-Coulter, Brea, CA) high speed flow cytometer/sorter. DAPI fluorescence was excited with the UV laser (355-nm), AlexaFluor 488, DCF and Rh123 with the argon ion (488-nm) laser. Although berberine, one of the studied agents, is fluorescent [132] control experiments excluded the possibility that its fluorescence significantly contributed to analysis of the measured cells that could lead to a bias. Statistical evaluation of individual measurements (SD) was carried out assuming the Poisson distribution in evaluation of populations of cells in particular phases of the cell cycle. All experiments were repeated at least three times, representative data are presented.

### Western Blotting

TK6 cells were exposed to the investigated agents at concentrations as shown in Figs. 1 and for 4 h. The **c**ells were then collected and lysed by incubation on ice for 30 min in cold immuno-precipitation (RIPA) buffer, which contained 50 mM Tris, pH 7.4, 150 mM NaCl, 1 mM EDTA, 1% Triton X-100, 1% deoxycholate, 0.1 % SDS, 1 mM dithiothreitol (DTT) and 10 μl/ml protease inhibitor cocktail and 1% phosphatase inhibitor cocktail 3 (Sigma-Aldrich). The extracts were centrifuged and the clear supernatants were stored in aliquots at -80°C for further analysis. Protein concentrations of cell lysates were determined by Coomassie protein assay kit (Pierce, Rockford, IL) using BSA as standard. Aliquots of lysates (10 μg of protein) were resolved by 10% SDS-PAGE followed by western blot analysis. The primary antibody against total 4EBP1 (C-19) was purchased from Santa Cruz Biotechnology, Inc. (Santa Cruz, CA). The primary antibodies for mTOR-Ser2448^P^, total mTOR, RP-S6-Ser235/236^P^, Total RB-S6, and 4EBP1-Ser65^P^ were obtained from Cell Signaling Technology, Inc. (Beverly, CA). The blots were first incubated with specific primary antibodies followed by secondary antibodies. Specific immunoreactive bands was identified and detected by enhanced chemiluminescence (ECL) using protocol provided by the manufacturer (Kirkegaard & Perry Laboratories, Inc., Gaithersburg, MD). The expression of actin was monitored in parallel as loading control. The intensity of specific immunoreactive bands was quantified by densitometry and expressed as a ratio relative to the expression of actin [133].
